# Microstructure and Mechanical Properties of Fe-30Mn-10Al-3.3Si-1C Light-Weight Steel

**DOI:** 10.3390/ma18061258

**Published:** 2025-03-12

**Authors:** Alena A. Kazakova, Alexander Yu. Churyumov

**Affiliations:** Department of Physical Metallurgy of Non-Ferrous Metals, National University of Science and Technology MISIS, Leninskiy Prospekt 4, 119049 Moscow, Russia; kazakova.aa@misis.ru

**Keywords:** high-Mn steel, hot deformation, Gleeble 3800, microstructure, mechanical properties, finite element simulation

## Abstract

The development of new materials with low weight for the transport industry is required for the saving of natural resources and protection of the environment from carbon dioxide pollution. The microstructure and mechanical properties of the Fe-30Mn-10Al-3.3Si-1C steel in as-cast, quenched, aged, and hot-deformed states were investigated. Austenite, ferrite, and κ-carbides are present in the steel in an as-cast state. Hot deformation of steels was made using the thermal and mechanical simulation system Gleeble-3800 at temperatures of 900–1050 °C and strain rates of 0.1–10 s^−1^. Mechanical properties in as-cast, annealed, aged, and hot-deformed states were determined by Vickers hardness and compression tests. A constitutive model of the hot deformation behavior of Fe-30Mn-10Al-3.3Si-1C steel with high accuracy (R^2^ = 0.995) was constructed. The finite element analysis of the deformation behavior of the steel under the plane-strain scheme was performed. Compression tests at room temperature have shown an increase in strength and ductility after hot deformation. The strain hardening of ferrite and austenite grain refinement during dynamic recrystallization are the main reasons for the growth of steel’s plasticity and strength. A specific strength of the investigated material is in the range from 202,000 to 233,000 m^2^/s^2^ which is higher than high-strength steels previously developed and used in the automotive industry.

## 1. Introduction

The requirements for steels constantly grow due to competition with non-ferrous metal alloys and non-metallic materials. Investigators should improve such contradictory properties as strength and ductility, as well as reduce the specific weight of steel. High-Mn steels are promising alloys for Fe-Cr-Ni steel replacements in the transport industry due to their elevated specific strength and relatively low cost due to the absence of expensive Ni. Such steels have high ductility, strength, and impact strength at low temperatures [1,2,3,4,5]. This type of steel is also useful for applications at cryogenic temperatures [6] and as a material for additive manufacturing [7]. Replacing mild steel with high-manganese steels significantly reduces the thickness of the front body sheet of automobiles, providing the same or better energy absorption capacity for impact protection [8,9] which may play an important role during automotive accidents on roads.

High-manganese-containing iron-base alloys usually have a stable austenitic microstructure at elevated temperatures and become metastable upon rapid cooling. The temperature range in which austenite exists in Fe-Mn-Al-C alloys increases with increasing Mn content up to 20% and narrows with further increase to 30%. The minimum temperature at which austenite exists as a stable single phase decreases to about 386 °C with increasing Mn content up to 30% [10]. Carbon is an essential component of steel. The purpose of carbon addition to alloyed steels is to strengthen them by forming carbon-supersaturated martensite during quenching and carbides during aging. The addition of 1 wt.% C to Fe-30Mn-9Al steel causes the steel structure to be completely austenitic [11].

The addition of aluminum increases the stability of ferrite and strengthens high-manganese steels with an austenitic microstructure. The addition of more than 2 wt.% aluminum promotes the appearance of spinodal decomposition, which results in the formation of supersaturated and unsaturated solid solutions of austenite [10,12]. Increasing the aluminum content helps to increase the stability of κ-carbides at high temperatures.

The mechanical properties of steel are mainly determined by deformation and heat treatment. One of the main technological processes is hot deformation. A study of Fe-27Mn-11.5Al-1C-0.6Si-0.043Nb steel during hot deformation [13] showed that at the initial stage of deformation, the dislocation density does not significantly increase. Basically, processes of strain hardening and recovery, as well as recrystallization, occur in ferrite. As the strain increases, the deformation propagates to the austenite grains. An important material parameter during the hot deformation is the effective activation energy. Early studies [14,15] showed that alloying with aluminum increases the activation energy of hot compression of high-manganese steel having a single-phase austenitic microstructure from 377 kJ/mol at 0% Al to 405 kJ/mol at 6% Al, but in the case of the appearance of ferrite at 8% Al, the activation energy decreased significantly to 300 kJ/mol, which indicates easier hot deformation.

In high-manganese content Fe-Mn-Al-C steels, deformation can occur by a variety of mechanisms. In the initial stage, the main mechanism of plastic deformation is dislocation slip, the formation of cellular or subgrain dislocation structures. Due to a decrease in stacking fault energy to the value of 20–40 mJ/m^2^, further cold deformation activates twinning and, as a result, refinement of the microstructure [16].

Fe-Mn-Al-C steels with a high content of Mn and Al can be strengthened by numerous precipitates, such as κ-carbide in austenite, B2, DO_3_ in ferrite, and β-Mn. Aging hardening is possible for Fe-30Mn-xAl-1C steels with Al content more than 8 wt. % [17]. The yield strength and ultimate tensile strength of Fe-30.4Mn-8Al-1.2C steel in a quenched state were 550 MPa and 1440 MPa, respectively [18]. After aging hardening with κ-carbides, these properties increased to 1020 MPa and 1510 MPa, respectively [19], while ductility decreased from 60% to 36%. The addition of 9% aluminum to Fe-20Mn-1.2C steel increases the yield strength from 360 MPa to 500 MPa in the annealed state and to 940 MPa in the aged state. The tensile strength decreased from 840 MPa to 715 MPa in the annealed state and increased from 500 MPa to 1015 MPa in the aged state [20]. Aging of steel without Al led to the formation of pearlite at the grain boundaries, while hardening of steel with the highest Al content was associated with the formation of fine dispersed κ-carbides. Steel with TRIPLEX (austenite + κ-carbide + ferrite) structure Fe-28Mn-12Al-1C has good ductility and strength at a wide temperature range [21].

The purpose of this research is to investigate the microstructure and mechanical properties of the high specific strength steel based on the Fe-Mn-Al-C system additionally alloyed with Si. Previously, high-Mn steels with an Si content of up to 2 wt.% were studied [22]. In the current work, we have investigated the influence of larger Si concentrations on the microstructure and mechanical properties of the steel.

## 2. Materials and Methods

**Figure 1:**
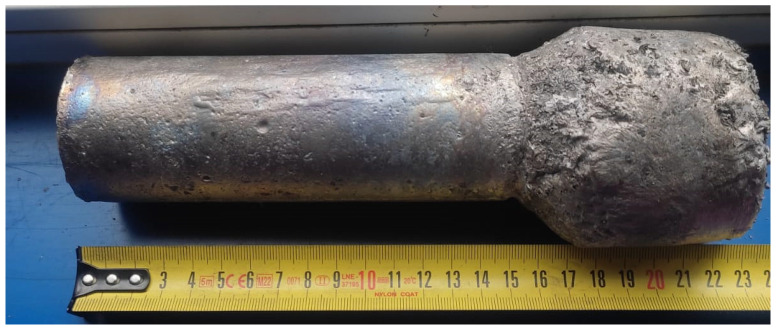
Ingot of Fe-30Mn-10Al-3.3Si-1C steel.

An ingot of Fe-30Mn-10Al-3Si-1C with a diameter of 50 mm, a length of 150 mm, and a weight of 3.5 kg (Figure 1) was obtained using the induction melting method. The gravitational casting under an argon atmosphere in a graphite mold was used to obtain the ingot. Nominal chemical composition of the alloy was obtained by inductively coupled plasma spectroscopy and is as follows: Fe-29.6Mn-9.8Al-1.02C-3.3Si-0.07Cr-0.02Ni-0.005P-0.005S (wt.%).

**Figure 2:**
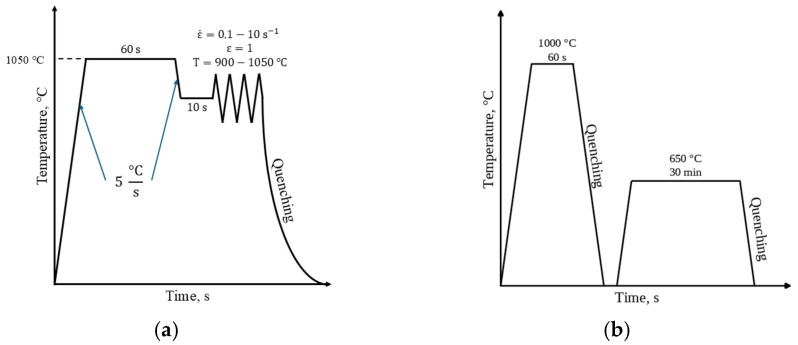
Hot deformation test (**a**) and thermal treatment (**b**) schemes.

Compression to a true strain of 1 was made using the Gleeble 3800 thermal and mechanical simulator (Gleeble, Poestenkill, NY, USA) at strain rates of 0.1, 1, and 10 s^−1^ and temperatures of 900–1050 °C with a step of 50 °C. The diameter and height of the samples were 10 mm and 15 mm, respectively. The samples were taken from the central part of the ingot (in the vertical direction) near the surface of the ingot. The samples were prepared using electrical discharge cutting. Graphite and tantalum foils were used to reduce the effects of the friction coefficient between the anvil and the edge of the specimen. Also, the primary stress–strain curves were corrected to take into consideration the adiabatic heating during compression accordingly [23]. The solution treatment and aging of the steel were performed in a vacuum furnace at temperatures of 1000 and 650 °C, respectively. After solution treatment, the samples were quenched in water to fix the high-temperature microstructure. The hot deformation test and thermal treatment schemes are presented in Figure 2.

**Figure 3:**
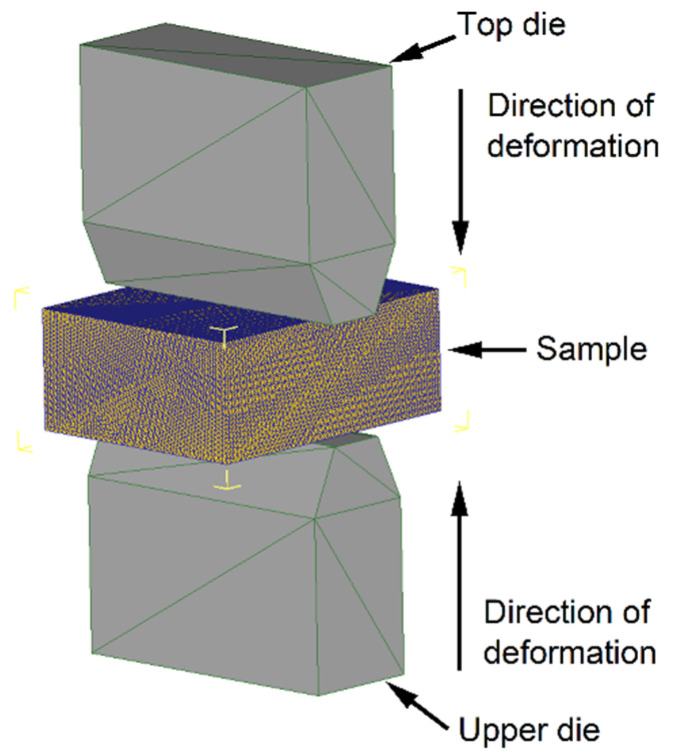
Test scheme for plane strain deformation.

To obtain samples for mechanical properties determination in the hot-deformed state, the plane strain deformation scheme was used (Figure 3). The size of the samples for deformation was 30 × 20 × 10 mm^3^. This scheme imitates the hot rolling scheme as the most common method of hot-forming sheet steel and makes it possible to obtain the same structure in the fractional transverse direction along the cross-section of the sample. The deformation was made using the Gleeble 3800 thermal and mechanical simulator.

Hardness measurement was carried out using the Wolpert MVD402 microhardness tester (ITW Test & Measurement GmbH, Leinfelden-Echterdingen, Germany) at a load of 500 g. Compression tests room temperature were performed using a Zwick Z250 (ZwickRoell, Atlanta, GA, USA) universal testing machine at a deformation rate of 4 mm/s. The size of the samples was 3 × 3 × 6 mm^3^. Three samples of each state were tested for data reproducibility. Sample mass density was measured using the hydrostatic weighing method.

The images of the microstructures were taken from the deformed cylindrical samples from a place located at 1/3 of the height and 1/3 of the width of the sample section. Metallographic samples were obtained using a grinding and polishing machine (Struers LaboPol-5, Struers, Champigny sur Marne, France). The samples were put into polystyrene; their surface was processed using sandpaper of different grain sizes (120–4000), then polished using a water-alcohol suspension of SiO_2_ and subjected to chemical etching in a 5% solution of HNO_3_ nitric acid in alcohol. A Zeiss Axiovert light microscope, a Tescan-VEGA3LMH scanning electron microscope (SEM) (Tescan, Brno, Czech Republic) with an energy dispersive X-ray X-MAX80 spectrometer (EDS) and a NordlysMax EBSD-HKL detector (EBSD) (Artisan, Champaign, IL, USA), and a Bruker Advance D8 X-ray diffractometer (Bruker, Billerica, MA, USA) were used for the analysis of the microstructure.

Automatic indexing for EBSD analysis used the Mean Angular Deviation (MAD); the MAD coefficient was 0.5. The results of the structure parameter studies using the EBSD analysis method were processed using the HKL Channel5 Oxford Instruments software v. 5.12, which includes the Mambo, Tango, and Salsa modules. The recrystallized grains were determined by the misorientation angle larger than 15 °.

Finite element analysis of the plane strain compression was made using Deform 3D software v. 11.0. The number of tetragonal elements in the mesh was 100,000. One point in each tetrahedral element was used for Gauss integration. The heat transfer and friction coefficients’ values between the sample and dies were chosen as 5 N/(s·mm·K) and 0.3, respectively. The type of sample and die was chosen as plastic and rigid, respectively. The shape of the dies and sample models is shown in Figure 3.

## 3. Results and Discussion

### 3.1. Microstructure and Phase Composition

**Figure 4:**
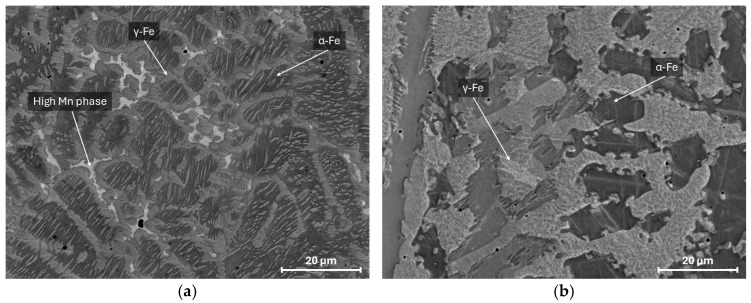
SEM microstructure of the investigated steel in as-cast (**a**) and solutioned (**b**) states.

**Figure 5:**
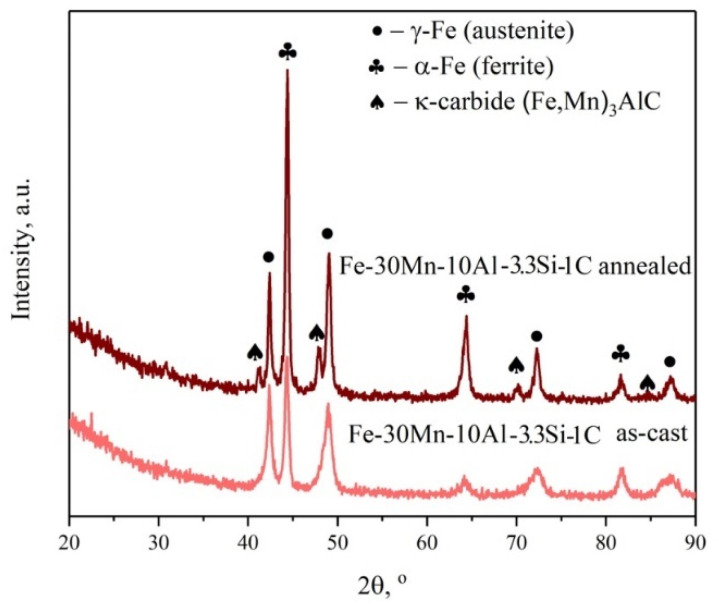
X-ray diffraction pattern of the investigated steel in as-cast and annealed states.

Figure 4a,b shows images of the microstructure of Fe-30Mn-10Al-3.3Si-1C steel in an as-cast and solutioned at 1000 °C state. The microstructure of steel mainly contains two phases: dark gray ferrite and light gray austenite. In addition, the structure contained a phase (white particles) with an increased content of manganese and a reduced concentration of aluminum. Presumably, this phase is a Mn-based solid solution. Solutioning leads to the full dissolution of the high-Mn phase and increases the austenite volume fraction. In addition, as seen in the XRD pattern (Figure 5), κ-carbides appeared in the microstructure after the solution treatment. As shown previously by Wang et al. [22], the formation of the intragranular κ-carbides may be accelerated in high-Mn steel alloyed by Si.

### 3.2. Hot Deformation

**Figure 6:**
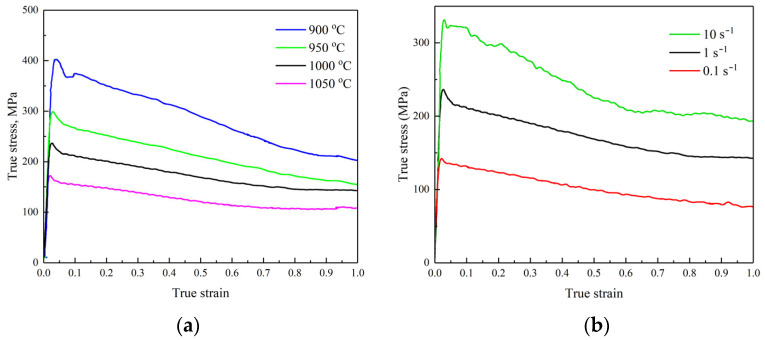
Compression curves of semi-industrial steel at the strain rate of 1 s^−1^ (**a**) and temperature of 1000 °C (**b**).

To analyze the behavior of the investigated steel during hot deformation, hot compressing experiments were performed. The typical true stress–true strain dependences are given in Figure 6. All curves have a maximum at the beginning of the deformation due to the start of the process of dynamic recrystallization. The peak is shifted to larger values of both strain and stress with decreases in the temperature and increases in the strain rate. It is the usual behavior for metallic materials and may be explained by the necessity of more time for the non-conservative dislocation movement for the new grains’ nucleation [24].

The deformation behavior at elevated temperatures of the metallic materials may be described by effective activation energy (*Q*). Its value is usually determined by usage of the dependence between true stress and deformation parameters using the universal Zener-Hollomon parameter (*Z*), which considers deformation rate and temperature:

(1) $$Z=\dot{\varepsilon }e^{\frac{Q}{RT}},$$

where $\dot{\varepsilon }$ is the strain rate (s^−1^), *T* is the deformation temperature (K), and *R* is a universal gas constant (8.314 J/molK). Under different deformation conditions, relations may be power (2), exponential (3), and hyperbolic sine (4):

(2) $$Z=A_{1}\sigma^{n_{1}}\;(\mathrm{for}\;\mathrm{low}\;\mathrm{values}\;\sigma )$$

(3) $${Z=A}_{2}e^{\beta \sigma }\;(\mathrm{for}\;\mathrm{high}\;\mathrm{values}\;\sigma )$$

(4) $$Z=A_{3}{\left[\sinh(\alpha \sigma )\right]}^{n_{2}}\;(\mathrm{for}\;\alpha \;\mathrm{values}\;\mathrm{in}\;\mathrm{the}\;\mathrm{all-deformation}\;\mathrm{range})$$

where *A*_1_, *A*_2_, *A*_3_, *n*_1_, *n*_2_, *β* and *α* are the specific steel constants. The coefficient *α* is related to the material constants *n*_1_ and *β* by the equation:

(5) $$\alpha \approx \frac{\beta }{n_{1}}$$

The steel constants were obtained using the least squares method from the peak true stress values. The dependence between peak stress and thermomechanical parameters may be described by the following equations:

(6) $$3.2\times 10^{16}{\left[\sinh(0.0035\sigma )\right]}^{4.2}=\dot{\varepsilon }e^{\frac{400,000}{RT}},$$

(7) $$\sigma =285.7\mathrm{asinh}[{0.31\times 10^{-16}{\dot{\varepsilon }e}^{\frac{400,000}{RT}}]}^{0.24}$$

**Figure 7:**
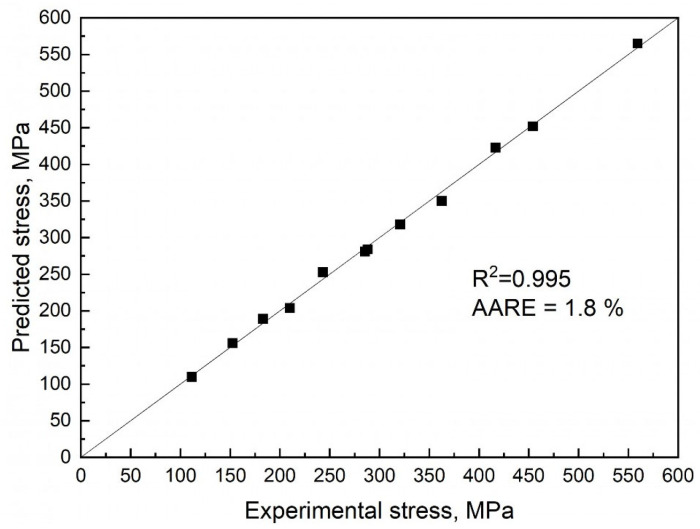
Comparison of predicted and experimental peak values of true stress during hot plastic deformation.

A comparison of predicted and experimental values of flow stress is presented in Figure 7. The average prediction error was 1.8%. The effective activation energy of hot plastic deformation for Fe-30Mn-10Al-3.3Si-1C was 400 kJ/mol, which is similar to the previously investigated high-Mn steels (Table 1).

**Figure 8:**
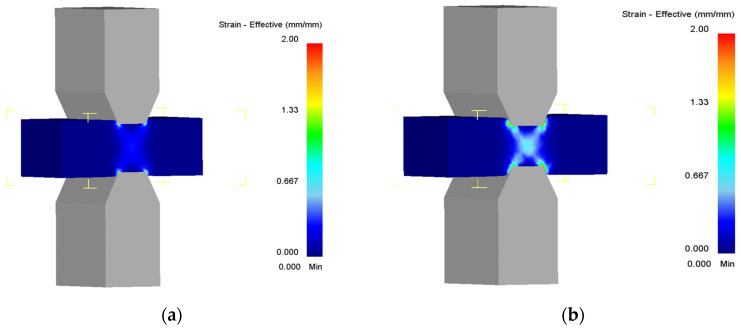

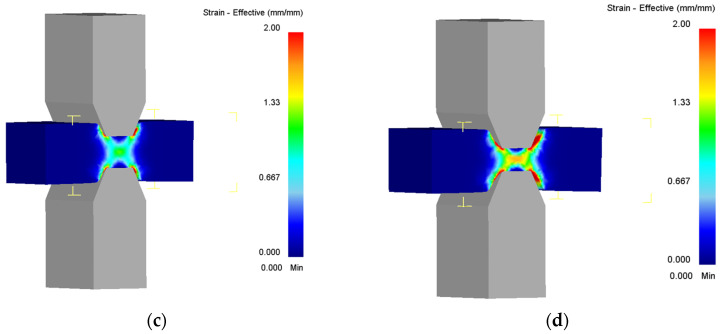
Distribution of strain over the cross-section of the sample during plane strain compression at the temperature of 1000 °C and at the strain rate of 0.1 s^−1^ at the strain of 0.25 (**a**), 0.5 (**b**), 0.75 (**c**), and 1 (**d**).

The obtained data were used for the finite element analysis of the hot plane strain compression. Figure 8 shows the distribution of strain over the cross-section of the sample at different stages of the compression. A significant difference in the strain values in the center of the sample and the place of contact with the dies is seen due to friction. However, the value of the strain is about 0.7, which corresponds to steady-state deformation according to compression curves. It means that the microstructure in the center and edges of the sample should not have significant differences.

**Figure 9:**
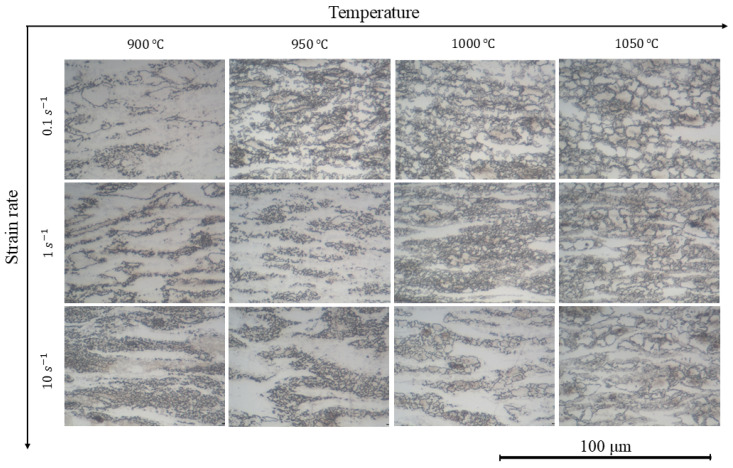
Microstructure of the investigated steel after the hot deformation.

The microstructure of the material after the deformation at various strain rates and temperatures is shown in Figure 9. The elongated initial grains are seen after the deformation at the temperature of 900 °C for all strain rates. The main recovery mechanism at this temperature is dynamic recovery. The increase in the compression temperature provides the possibility for the appearance of the nuclei of new grains and their growth. The microstructure becomes more uniform at higher temperatures, which is associated with a more complete dynamic recrystallization, mainly in austenite phases with the strain rate decrease. The maximum grain size is after the deformation at 1050 °C and a strain rate of 0.1 c^−1^.

**Figure 10:**
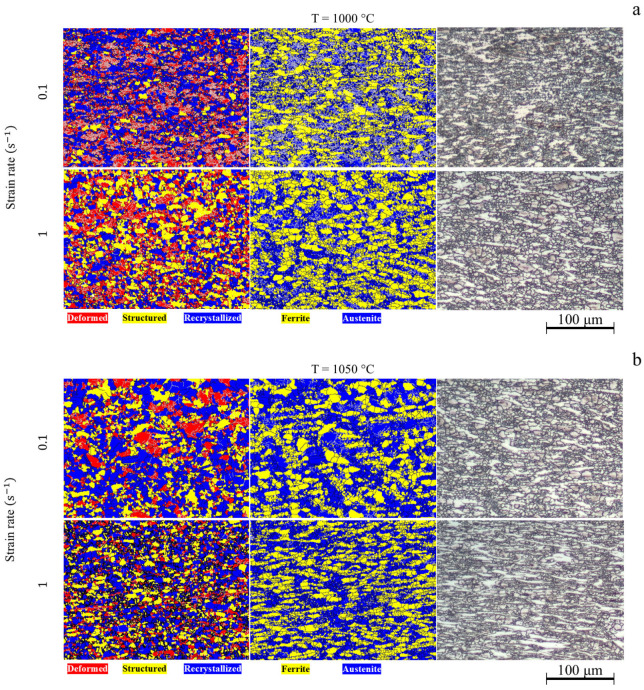
Microstructure of the investigated steel after deformation at (**a**) 1000 °C and (**b**) 1050 °C.

**Figure 11:**
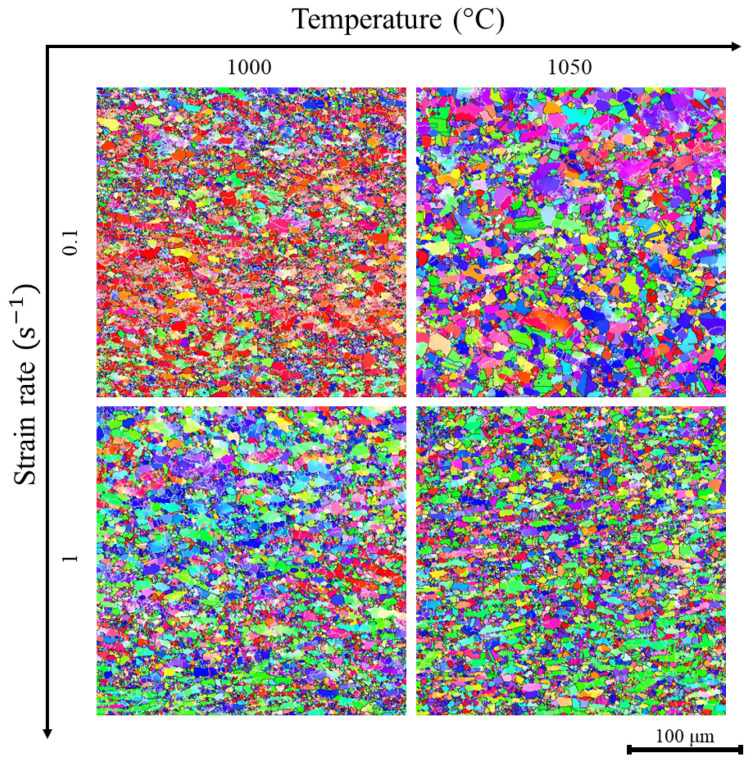
EBSD maps of grain misorientation of the investigated steel after deformation.

EBSD analysis of the steel showed that after hot deformation, the structure contains recrystallized austenite grains and deformed ferrite grains (Figure 10). At a temperature of 1000 °C, non-recrystallized grains predominate in the microstructure. The volume fraction of recrystallized grains increases when the temperature increases to 1050 °C due to the acceleration of the non-conservative movement of dislocations providing more centers of the dynamic recrystallization. The average grain size of both phases does not significantly depend on the strain rate of 1 s^−1^ (Figure 11). However, the grain size increases significantly from 7 ± 0.4 μm at 1000 °C to 19 ± 2 μm at 1050 °C at a lower strain rate. The accelerated grain growth proceeds due to the high mobility of the dislocations at higher temperatures. As a result, the number of nuclei of the dynamically recrystallized grains decreased. In addition, the mobility of the grain boundaries is also higher at elevated temperatures due to accelerated diffusion. Similar outputs were obtained by P. Zhang et al. for high-Mn steel [30] and T. Zhang for Fe-Mn-Al-C steel [31].

### 3.3. Mechanical Properties

**Figure 12:**
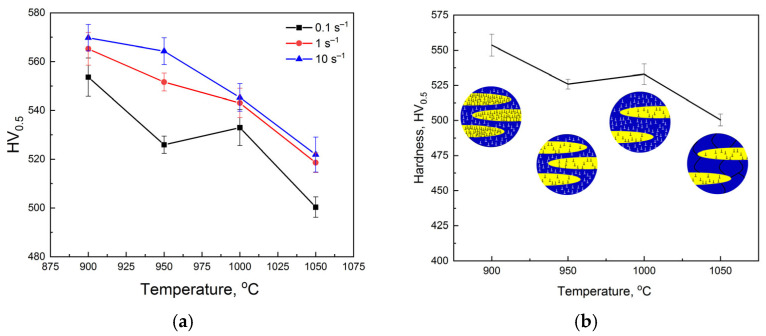
Hardness of Fe-30Mn-10Al-3.3Si-1C steel after hot plastic deformation (**a**) and possible mechanism of the microstructural changes during the hot deformation (**b**) (blue phase is austenite, yellow phase is ferrite).

The values of steel hardness are given in Table 2 and Table 3. As seen, the hardness increases after the quenching and aging due to the dissolution of the coarse carbides during solutioning and the appearance of dispersed carbides during aging. The hardness of the steel is significantly determined by the thermomechanical conditions (Figure 12a). The rise in the strain rate and reduction in the temperature increase the hardness. However, the decrease in hardness with deformation temperature has a non-monotonic tendency for the strain rate of 0.1 s^−1^. The hardness has a minimum at 950 °C. The scheme presented in Figure 12b is proposed to explain the decrease in hardness (austenite is marked in blue, ferrite in yellow). In the case of deformation at a temperature of 900 °C, austenite and ferrite are in a deformed state with an elevated dislocation density. An increase in the temperature to 950 °C provides intensive dynamic recovery in ferrite, which leads to a decrease in overall hardness due to a large volume content of ferrite. The amount of deformed austenite at 1000 °C leads to increases in the hardness. At higher temperatures, both phases are softened due to dynamic recovery in ferrite and dynamic recrystallization in austenite.

**Figure 13:**
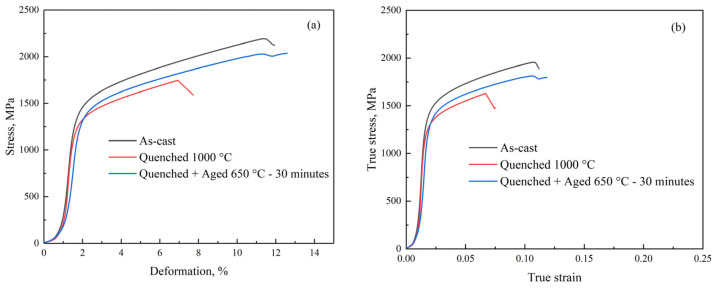
Compression diagrams of semi-industrial steel in as-cast, quenched, and quenched and aged states. Engineering stress vs. deformation (**a**) and true stress vs. true strain (**b**).

Compression curves of the investigated steel in the cast, annealed at 1050 °C and quenched state, as well as in the quenched and aged state, are presented in Figure 13. As seen, the material shows significant constant strain hardening up to fracture (Figure 13b). The strain hardening is independent of the state of the steel and has a value of about 43 MPa/%. The mechanical properties of the steel are given in Table 2. The yield stress is almost on the same level for all states. However, the quenching decreases the ductility of steel, which may be associated with residual stresses [32]. Specific strength was calculated using mass density 6.47 g/sm^3^ obtained using the formula for Fe-Mn-Al-C alloys [33]. Experimental results have confirmed that value.

**Figure 14:**
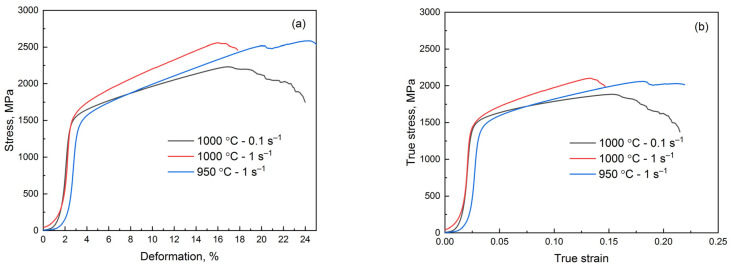
Compression diagrams of hot-deformed under different conditions semi-industrial steel. Engineering stress vs. deformation (**a**) and true stress vs. true strain (**b**).

The typical stress–strain curves and mechanical properties of the investigated steel in the hot-deformed state are given in Figure 14 and Table 3. It can be seen that the hot plastic deformation significantly increases the ductility of the steel. The maximum strength of 1510 MPa was obtained after hot deformation at an increased strain rate. An increase in the strain rate does not fully provide the dynamic recovery in the ferrite and austenite. As a result, the difference in the strength after the deformation at 1000 °C at low and high strain rates is about 50 MPa. In addition, the grain refinement of austenite during the dynamic recrystallization also contributes to the strength of the steel. As seen, the elongated initial grains that remain after the deformation at 950 °C do not make a significant contribution to the strength.

**Figure 15:**
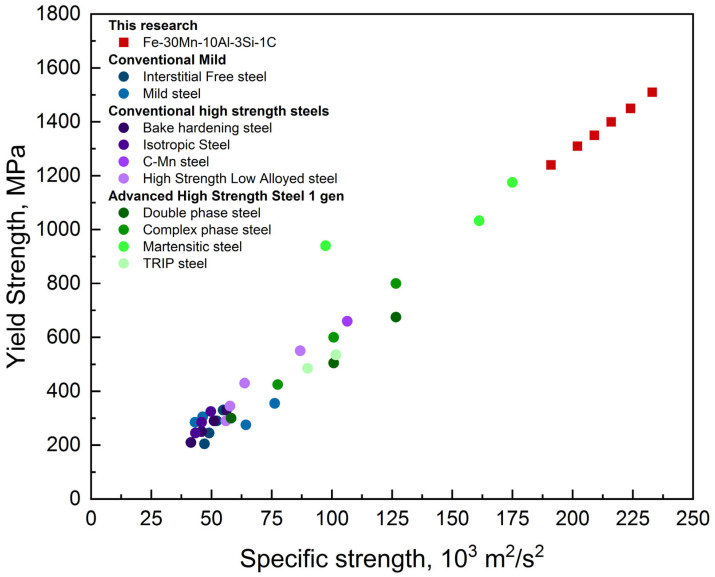
A comparison of the automotive steels’ specific strength (the data for steels for comparison was taken from European Standard EN 10025:2004).

The investigated steel shows a high specific strength of 202,000–233,000 m^2^/s^2^, which is significantly higher than the strength of the steels used currently for automotive parts according to European standard EN 10025:2004 [34] (Figure 15).

## 4. Conclusions

1. The microstructure and mechanical properties of the Fe-30Mn-10Al-3.3Si-1C steel in as-cast, quenched, aged, and hot-deformed states were investigated. Austenite, ferrite, and κ-carbides are present in the steel in an as-cast state. It was shown that annealing at a temperature of 1050 °C leads to full dissolution of κ-carbides.
2. A constitutive model of the hot deformation behavior of the investigated material was constructed:

$$3.2\times 10^{16}{\left[\sinh\left(0.0035\sigma \right)\right]}^{4.2}=\dot{\varepsilon }e^{\frac{400,000}{RT}}$$

It was found that the effective activation energy of the steel has a similar value (400 kJ/mol) to the values obtained for one-phase austenitic Fe-Mn-Al-C steels.

3. The hardness of the steel has values in the range of 500–580 HV, with a maximum after the hot deformation at a high strain rate and low temperatures. The minimum hardness in the temperature dependence for a 0.1 s^−1^ strain rate may be described by the competitive processes of phase transformation and dynamic softening.
4. Compression tests showed an increase in strength and ductility after the hot deformation. The specific strength of the steel has values of 202,000–233,000 m^2^/s^2^, which is higher than currently used automotive steels.

## Figures and Tables

**Table 1:** The effective activation energy of the hot deformation of Fe-Mn-Al-C steels.

Steel	Phase Composition at Deformation Temperatures	Effective Activation Energy of Hot Deformation
Fe-30Mn-10Al-3.3Si-1C [this work]	Austenite/Ferrite	400 ± 13 kJ/mol
Fe-28Mn-8.8Al-0.9C [25]	Austenite	394 kJ/mol
Fe-28Mn-8Al-1C [26]	Austenite	385 kJ/mol
Fe-35Mn-10Al-1C [27]	Austenite	432 kJ/mol
Fe-30Mn-11Al-1C-0.1Nb-0.1V [28]	Austenite	389 kJ/mol
Fe-25Mn-10Al-1.5C-0.053Nb [29]	Austenite	513 kJ/mol

**Table 2:** Mechanical properties of the steel after different heat treatments.

State	Yield Strength, MPa	True Compressive Strength, MPa	Deformation to Fracture, %	Specific Strength, σ_0.2_/ρ, 10^3^ m^2^/s^2^	Vickers Hardness, HV0.5
As-cast	1350 ± 32	1960 ± 45	8 ± 2	209 ± 5	508 ± 3
Quenched at 1000 °C	1350 ± 35	1620 ± 41	5 ± 1	209 ± 5	530 ± 16
Quenched at 1000 °C + aging at 650 for 30 min	1310 ± 25	1810 ± 35	8 ± 1	202 ± 4	550 ± 17

**Table 3:** Mechanical properties of steel in the hot-deformed state.

State	Yield Strength, MPa	True Compressive Strength, MPa	Deformation to Fracture, %	Specific Strength, σ_0.2_/ρ, 10^3^ m^2^/s^2^	Vickers Hardness, HV0.5
1000 °C—0.1 s^−1^	1450 ± 41	1880 ± 52	13 ± 2	224 ± 6	532 ± 12
1000 °C—1 s^−1^	1510 ± 38	2100 ± 45	13 ± 2	233 ± 6	543 ± 10
950 °C—0.1 s^−1^	1400 ± 35	2060 ± 54	16 ± 3	216 ± 5	526 ± 8

## Data Availability

The original contributions presented in this study are included in the article. Further inquiries can be directed to the corresponding author.
